# Exploring the Midgut Transcriptome and Brush Border Membrane Vesicle Proteome of the Rice Stem Borer, *Chilo suppressalis* (Walker)

**DOI:** 10.1371/journal.pone.0038151

**Published:** 2012-05-29

**Authors:** Weihua Ma, Zan Zhang, Chuanhua Peng, Xiaoping Wang, Fei Li, Yongjun Lin

**Affiliations:** 1 National Key Laboratory of Crop Genetic Improvement and National Centre of Plant Gene Research, Huazhong Agricultural University, Wuhan, China; 2 Hubei Insect Resources Utilization and Sustainable Pest Management Key Laboratory (Huazhong Agricultural University), Wuhan, China; 3 Department of Entomology, Nanjing Agricultural University; Key Laboratory of Monitoring and Management of Plant Diseases and Pests, Ministry of Agriculture, Nanjing, China; 4 Hubei Plant Protection Station, Wuhan, China; Cinvestav, Mexico

## Abstract

The rice stem borer, *Chilo suppressalis* (Walker) (Lepidoptera: Pyralidae), is one of the most detrimental pests affecting rice crops. The use of *Bacillus thuringiensis* (Bt) toxins has been explored as a means to control this pest, but the potential for *C. suppressalis* to develop resistance to Bt toxins makes this approach problematic. Few *C. suppressalis* gene sequences are known, which makes in-depth study of gene function difficult. Herein, we sequenced the midgut transcriptome of the rice stem borer. In total, 37,040 contigs were obtained, with a mean size of 497 bp. As expected, the transcripts of *C. suppressalis* shared high similarity with arthropod genes. Gene ontology and KEGG analysis were used to classify the gene functions in *C. suppressalis*. Using the midgut transcriptome data, we conducted a proteome analysis to identify proteins expressed abundantly in the brush border membrane vesicles (BBMV). Of the 100 top abundant proteins that were excised and subjected to mass spectrometry analysis, 74 share high similarity with known proteins. Among these proteins, Western blot analysis showed that Aminopeptidase N and EH domain-containing protein have the binding activities with Bt-toxin Cry1Ac. These data provide invaluable information about the gene sequences of *C. suppressalis* and the proteins that bind with Cry1Ac.

## Introduction

The rice stem borer, *Chilo suppressalis* (Walker) (Lepidoptera: Pyralidae), is one of the most important rice pests. Various methods have been used to try to control *C*. *suppressalis*, including cultural practices, pheromone traps, planting resistant rice varieties, and insecticides. Among these methods, farmers still prefer to use insecticides because of their high efficiency [1]. However, widespread insecticide use poses potential threats to the environment and food safety. Thus, an alternative strategy for controlling the rice stem borer is needed. One promising possibility is use of the toxic protein produced by the bacterium *Bacillus thuringiensis* (Bt). Bt toxins are considered to be safe and have been widely used in transgenic plants to control lepidopteran pests such as *Helicoverpa armigera* [2]–[5]. Although this tactic is successful in controlling rice stem borer at present, the ability of target pests to develop resistance poses a challenge. Better understanding of the interactions between Bt toxins and the receptors in the midgut of pests is very important for monitoring resistance and for pest management [6]–[11].

Despite the economic importance of the damage caused by *C. suppressalis*, genetic information about this species is scarce. Few *C. suppressalis* gene sequences are known: Currently, only 192 annotated genes and 147 protein sequences are available in the NCBI database. The lack of gene sequences hinders in-depth study of gene function in *C*. *suppressalis*. The midgut of insect pests is an important organ because it participates in digestion, detoxification, and nutrient intake. It is also the place where Bt toxins interact with insect receptors. Therefore, exploring the midgut transcriptome and proteome of *C. suppressalis* will provide invaluable information about its gene sequences and promote investigations of plant-insect interactions.

To gain insight into the complexity of the midgut transcriptome and proteome of *C*. *suppressalis* and to identify genes and proteins related to digestion and detoxification, we conducted high-throughput Illumina sequencing of the midgut transcriptome of *C*. *suppressalis* and two-dimensional protein electrophoresis in combination with mass spectrometry of the brush border membrane vesicle (BBMV) proteome. This analysis dramatically increases the number of known genes and proteins of midgut and enhances the understanding of the *C. suppressalis* midgut.

## Results and Discussion

### Illumina sequencing

The *C. suppressalis* midgut cDNA sample was prepared and sequenced using the Illumina sequencing platform. After cleaning and quality checks, we obtained 39 million 90-bp-long reads,which were submitted to the SRA database with accession number SRA050703.2. After they were assembled using Trinity [12] software, a total of 37,040 contigs were obtained (Table 1). The mean contig size was 497 bp, with lengths ranging from 201 to 9,744 bp. The size distribution of these contigs is shown in Figure S1.

**Table 1:** Summary for the *Chilo suppressalis* midgut transcriptome.

Total number of reads	39,400,002
Total base pairs (bp)	3,546,000,360
Total number of contigs	37,040
Mean length of contigs (bp)	497
Sequences with E-value <10^−5^	15,446
GC percentage	42%

### Annotation of predicted proteins

Among the midgut transcripts, 15,446 (41%) showed significant similarity (E-value <1e^−5^) to known proteins in the NCBI database (Table 2). The majority of the transcripts with similar sequences in the database (35%) matched to arthropod proteins. The remaining midgut transcripts were similar to proteins of non-insect eukaryotes (3%) and bacteria (3%). A total of 132 sequences were similar to viral proteins, and one midgut sequence matched to an Archaea protein (Table 2).

**Table 2:** Summary of BLASTX search of the *Chilo suppressalis* midgut sequences.

Significant matches	15,446
Archaea	1
Arthropoda	12,935
Bacteria	1,224
Other eukaryotes	1,154
Viruses	132
Non-significant matches	21,594
Total	37,040

### Comparative analysis

**Figure 1:**
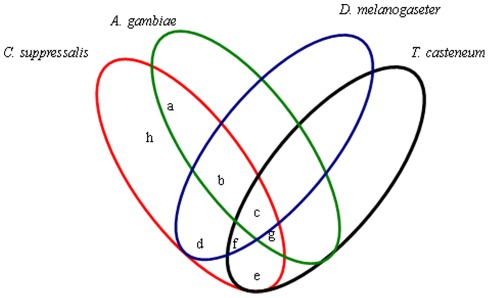
Summary of the comparisons analysis. *Chilo suppressalis* transcriptomic sequences were used to compare with protein sequences from the draft genomes of *Anopheles gambiae*, *Drosophila melanogaster*, and *Tribolium casteneum*. a=228; b=e; c=11419; d=138; e=1564; f=435; g=550; h=22320.

Comparison of the derived *C. suppressalis* midgut transcripts with protein sequences in the draft genomes of *Drosophila melanogaster*, *Anopheles gambiae*, and *Tribolium casteneum* conducted using the BLASTX algorithm program revealed high sequence similarity (38%, 13,968 out of 37,040) to the *T. casteneum* genome (Figure 1). The analysis also showed 33% and 34% similarity to the genomes of *D. melanogaster* and *A*. *gambiae*, respectively. A total of 11,419 sequences were shared among all four insect species. About 60% of sequences (22,320 out of 37,040) did not show BLASTX similarity, implying that they represented un-translated regions, non-conserved regions, or proteins novel to *C. suppressalis* (Figure 1).

### COG classification

**Figure 2:**
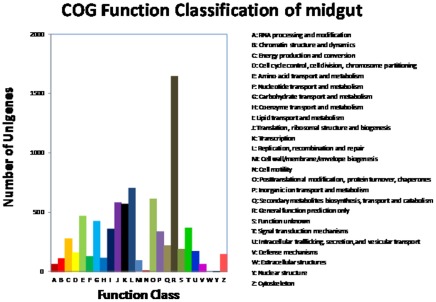
Histogram presentation of clusters of orthologous groups classification. Out of 345,905 nr hits, 10,967 sequences have a COG classification among the 25 categories.

To further evaluate the completeness of our transcriptome library and the effectiveness of our annotation process, we searched the annotated sequences for the genes involved in COG classification. In total, out of 15,446 hits, 5,824 sequences have a COG classification (Figure 2). Among the 25 COG categories, the cluster for “general function prediction” represents the largest group (1,646, 28%), followed by “replication, recombination and repair” (704, 12%) and “translation, ribosomal structure and biogenesis” (584, 10%). The categories of “extracellular structures” (1, 0.01717%) and “nuclear structure” (2, 0.03434%) represent the smallest groups (Figure 2). Similar observations were reported by Wang et al. [13] for the transcriptome of the whitefly *Bemisia tabaci*. To identify the biological pathways that are active in the *C. suppressalis* midgut, we mapped the 15,446 annotated sequences to the reference canonical pathways in the Kyoto Encyclopedia of Genes and Genomes (KEGG) [14]. In total, we assigned 12,358 sequences to 218 KEGG pathways (Table S1). Among these sequences, 1,252 belong to metabolic pathways, followed by 373 sequences in spliceosome pathway and 246 sequences in purine metabolism.

### Protein domains

A Pfam domain search yielded 11,895 domains within the 37,040 contigs that have significant hits in the NCBI nr database (Table 3). Among the identified Pfam domains, zinc finger C2H2 type (Znf–C2H2), which is the most common DNA-binding motif [15], was the most abundant, existing in 542 transcripts (5%) from the *C. suppressalis* midgut. The WD G-beta repeat was the second most abundant domain in the *C. suppressalis* midgut sequences (Table 3). The domains related to reverse transcriptase, protein kinase, sugar transporters, carboxyl esterase, and trypsin also were abundant in these contigs (Table 3, Table S2). These results are consistent with a previous analysis of Pfam domains in *Agrilus planipennis* midgut [16], which may indicate the existence of similar gene profiles in the midgut of insects.

**Table 3:** Top Pfam domains identified in *Chilo suppressalis* midgut sequences.

Pfam accession	Pfam domain description	Number of occurrence in midgut
PF00096.20	Zinc finger, C2H2 type	542
PF00400.26	WD domain, G-beta repeat	394
PF00069.19	Protein kinase domain	152
PF00076.16	RNA recognition motif. (a.k.a. RRM, RBD, or RNP domain)	149
PF00078.21	Reverse transcriptase (RNA-dependent DNA polymerase)	130
PF07679.10	Immunoglobulin I-set domain	108
PF00135.22	Carboxylesterase family	99
PF00560.27	Leucine Rich Repeat	95
PF00435.15	Spectrin repeat	93
PF00089.20	Trypsin	84
PF00083.18	Sugar (and other) transporter	83
PF07690.10	Major Facilitator Superfamily	81
PF00153.21	Mitochondrial carrier protein	79
PF00067.16	Cytochrome P450	70
PF12796.1	Ankyrin repeats (3 copies)	70
PF00106.19	short chain dehydrogenase	62

### Genes of interest

In this study, we were interested in identifying genes involved in detoxification and the antioxidant response, Bt Cry1A toxin binding, digestion, immunity and defense, metabolism, remodeling and peritrophic membrane biosynthesis, (Table 4). Of particular interest are proteins that bind the Bt-toxin Cry1A, which are known to be cadherin-like proteins [17]–[22] and glycosylphosphatidylinositol (GPI)-anchored proteins (aminopeptidase N, APN, or alkaline phosphatase, ALP) [23]–[33]. We identified 16 cadherin-like proteins, 27 APNs, and 11 ALP proteins in the *C. suppressalis* midgut.

**Table 4:** Selection of genes of interest related to the larval midgut physiological functions.

Total number of contigs (nr annotation)	
‘Detox’ related:	
Cytochrome P450	63
Glutathione-S-transferase	14
Carboxylesterase	16
Superoxide dismutase	5
*Bacillus thuringiensis* Cry1A toxins binding partners:	
Cadherin-like	16
Aminopeptidase N	27
Alkaline phosphatase	11
Digestion:	
Serine proteinase all types	14
Cysteine proteinase all types	7
Carboxypeptidase all types	46
Aminopeptidase all types	33
Dipeptidyl-peptidase	5
α-amylase	10
α-glucosidase (maltase)	2
β-glucosidase	11
Lipase	51
Immunity-related and defence against	
β-1,3-glucan recognition protein	12
β-1,3-glucanase	1
Peptidoglycan recognition protein	4
Immuno- and C-type lectins	20
Defensin-like	1
Lysozyme	11
Serine protease inhibitor (Serpin)	39
Transferrin	7
Peritrophic membrane biosynthesis, metabolization and remodelling:	
Chitin synthase	3
Chitinase	11
Chitin deacetylase	10
Peritrophin-like, Mucin-like	19

### Exploring the BBMV proteome of *C. suppressalis*

**Figure 3:**
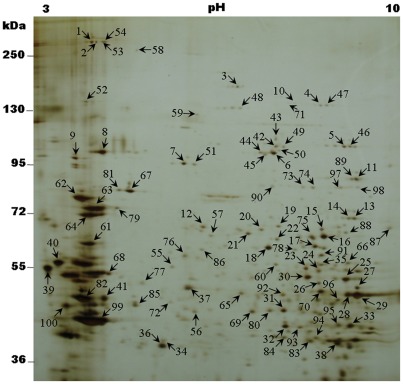
Representative example of 2D-gel of *Chilo suppressalis* larval midgut BBMV. BBMV were separated by 2D-gel electrophoresis followed by silver staining. Positions of molecular size markers (kDa) are indicated on the side of gel. Abundant protein spots were excised (numbers 1–100), digested, and subjected to mass spectrometry analysis for identification. Spot numbers correspond to the proteins listed in Table S3.

The most abundant proteins in the BBMV of *C. suppressalis* were also investigated in this study. Figure 3 shows a representative two-dimensional (2D) electrophoresis gel revealing the profile of BBMV proteins from *C. suppressalis*. Among the detected protein spots, 100 abundant proteins that were present on replicated gels were excised and subjected to mass spectrometry analysis. The spectra obtained from MALDI-TOF/TOF were searched for in a local database (translated transcriptome data) and the NCBInr database, and a total of 74 selected proteins had significant results (Table S3). The abundant proteins identified by this proteome analysis are consistent with the transcriptome data. The RNA transcripts encoding these proteins are also abundant in the transcriptome of the *C. suppressalis* midgut.

The annotations of the identified proteins from the UniProt knowledge base (http://www.expasy.org/sprot/) were used to categorize the proteins based on their molecular function in terms of information obtained from the GO database (http://www.geneontology.org/) (Table S3). The identified proteins were functionally categorized as follows: binding proteins, structural maintenance, metabolism and electron transport processes, translation factors, molecular transducers, transcription factors, antioxidant and redox processes, and digestion (Table S3). The proteins identified in this study are in good agreement with other proteomic analyses of the lepidopteran midgut [34]–[38], which indicates that a common protein complex is shared among lepidopteran insects.

### Western blot

**Figure 4:**
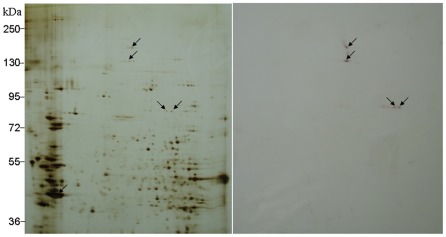
Blot analysis of Cry1Ac binding proteins in *Chilo suppressalis* midgut. Proteins were separated by 2D-gel electrophoresis. Arrows denote positions of the Cry1Ac binding proteins. To detect Cry1Ac binding proteins, filters were probed with biotin-Cry1Ac. Positions of molecular size markers (kDa) are indicated on the side of gel.

**Figure 5:**
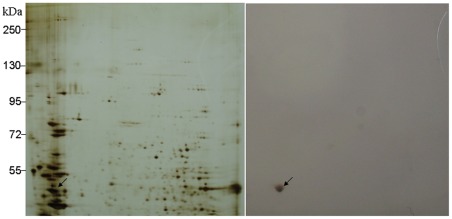
Blot detection of actin in *Chilo suppressalis* midgut. Proteins were separated by 2D-gel electrophoresis. Arrows denote positions of the anti-actin antibody binding protein. The filters were first probed with mouse anti-beta-actin monoclonal antibody (Sigma) and then visualized with goat anti-mouse IgG conjugated with alkaline phosphatase (Sigma) and the BCIP/NBT system (Sigma). Positions of molecular size markers (kDa) are indicated on the side of gel.

To verify the accuracy of our transcriptome and proteome sequences, we chose the Bt-toxin Cry1Ac binding protein and actin as the molecule markers. Western blot analysis was used to detect the proteins that bind Bt toxins in the midgut of *C. suppressalis*. As expected, the biotin-labeled Cry1Ac showed significant binding activity with spots 3, 48, 73, and 74 (Figures 3 and 4, Table S3). Using the MALDI-TOF/TOF technique, spot 3 and 48 were identified as two isoforms of APN. Spot 74 was identified as EH domain-containing protein 1, but spot 73 had no significant match (Table S3). These results confirmed that APN binds with the Cry1Ac toxin [39]. To the best of our knowledge, this is the first report that EH domain-containing protein 1 interacts with Bt toxins, and this result should be confirmed by further study. Unexpectedly, cadherin was not identified as a potential binding protein for Cry1Ac, which was reported previously [39]. We reasoned that the absence of cadherin in our proteome might be due to the limitation of 2D electrophoresis that is not suitable for separating proteins larger than ∼200 kDa. Actin is found in almost all eukaryotic cells and is widely used as a molecule marker in many kinds of molecular experiments. Spot 82 was identified as actin and showed strong binding activity to the mouse anti-beta-actin monoclonal antibody (Sigma) (Figures 3 and 5, Table S3).

## Materials and Methods

### Insect rearing and midgut sample preparation

The *C. suppressalis* strain was collected from the farm at the Huazhong Agricultural University, Wuhan, Hubei, China in 2010. This strain was reared in plastic containers (15 cm diameter ×8 cm high) on an artificial diet at 28±1°C under a 16 h photoperiod and >80% relative humidity. For total RNA and protein preparation, midguts were dissected from fifth instar larvae and cleaned in ice-cold 0.7% NaCl solution, immediately frozen in liquid nitrogen, and then stored at −80°C until use. The midgut content was removed before processing.

### RNA isolation and library preparation for transcriptome analysis

Total RNA was isolated using the SV total RNA isolation system (Promega) according to the manufacturer's protocol. To obtain complete gene expression information, the RNA sample from the midgut was used for transcriptome analysis. Poly(A) + RNA was purified using oligo(dT) magnetic beads and fragmented into short sequences at 94°C for 5 min. The cleaved poly(A) + RNA was transcribed, followed by synthesis of the second-strand cDNA. After the end repair and ligation of adaptors, the products were amplified by PCR and purified using the QIAquick PCR Purification Kit.

The cDNA library was sequenced on the Illumina sequencing platform (GAII). The raw reads from the images were generated using Solexa GA pipeline 1.6.

### Bioinformatics data analysis

The high quality reads were assembled into contigs using Trinity software [12]. All contigs were used as the queries to search a local protein database containing all of the protein sequences of the nr database with the BLASTX algorithm. COG classification was analyzed using annotated contigs to search the COG database with the cutoff 10^–3^. For KEGG pathway analysis, all contigs was used to search the KEGG database with default parameters. For comparative genomics analysis, the annotated contigs were used to BLASTP against known genes in three insect species: *Drosophila melanogaster*, *Anopheles gambiae*, and *Tribolium casteneum.*

### BBMV extraction and 2D SDS-PAGE Gel Analysis

The BBMV were prepared according to a published method [40]. Briefly, midguts were homogenized in ice-cold Tris/Mannitol buffer (300 mM mannitol, 5 mM EGTA, 17 mM Tris-HCl, pH 7.5) containing a cocktail of protease inhibitors (Roche) and 24 mM MgCl_2_. The homogenate was centrifuged at 2500 g at 4°C for 15 min, and the supernatant was centrifuged at 30,000 g at 4°C for 30 min. The pellet was resuspended in Tris/CHAPS buffer (150 mM NaCl, 5 mM EGTA, 1 mM PMSF, 20 mM Tris-HCl, 1% CHAPS) containing a cocktail of protease inhibitors (Roche).

BBMV proteins were processed using the 2D-Clean-up Kit (Bio-Rad) following the manufacturer's instructions. These proteins were resuspended in a solubilization buffer containing 2 M thiourea, 7 M urea, 4% CHAPS, 65 mM DTT, and 0.2% Bio-lyte ampholytes (pH 3–10, Bio-Rad). Final BBMV samples were centrifuged at 12,000 g, 4°C for 5 min to remove any insoluble material. Isoelectric focusing (IEF) was performed on a Protean IEF Cell (Bio-Rad). Midgut BBMV proteins were applied to 11 cm IPG strips at pH 3–10 (Bio-Rad) for separation. Equilibrated strips were overlaid onto a 10% SDS-polyacrylamide gel for the second dimension separation. Gels were visualized by silver staining [41].

### Mass spectrometric analysis and database searching

Stained protein spots were excised from the gel. Before MS/MS analyses, mixtures of proteolytic peptides were desalted using C18 ziptips. Samples were analyzed in the positive ion mode using a 4800 Plus MALDI-TOF/TOF TM Analyzer (Applied Biosystems), and MS/MS of the 10 most intense ions (with S/N >50 per digest) was analyzed using automated data acquisition. Spectra were processed and batch analyzed in the “Combined (MS + MS/MS)’’ mode using the Applied Biosystems GPS Explorer software.

The data were then used to search our local database of *C. suppressalis* midgut transcriptome sequences. MALDI-TOF/TOF data searches were also performed in the nr database (translated transcriptome sequences) using the MASCOT program. The search parameters used were as follows: “metazoa” was used for “taxonomy” and “trypsin” was used for “enzyme of specificity strict”; fixed modifications of carbamidomethyl (C); peptide tolerance of 100 ppm; fragment mass tolerance of 0.8 Da; peptide charge of 1+; and monoisotopic. Only significant hits (*p*<0.05) were accepted. Manual de novo sequence searching against the NCBI-BLASTP database, e-values, and frequency of matches to a specific protein were also the primary criteria for these determinations.

### Western and ligand blotting

Separated BBMV were transferred onto NC membranes at 15 V constant voltage for 1 h using the Trans-Blot® SD Semi-Dry Electrophoretic Transfer Cell system (Bio-Rad). Membranes were blocked with 5% skimmed milk in PBST (137 mM NaCl, 2 mM KCl, 10 mM Na_2_HPO_4_, 2 mM KH_2_PO_4_, pH 7.4, 0.05% Tween-20) for 2 h. Following this incubation, the membranes were washed with three changes of PBST for10 min each.

For detection of Cry1Ac binding proteins, the pure activated Cry1Ac toxins (EnviroLogix Inc.) were first marked with biotin (Roche). The blocked membranes were incubated with biotinylated Cry1Ac (1∶5000) for 1 h at room temperature. After washing, membranes were incubated for 1 h with a 1∶5000 dilution of streptavidin-alkaline phosphatase conjugate (Sigma). Binding proteins were visualized using the BCIP®/NBT Kit (Sigma) following the manufacturer's instructions.

For detection of actin, blocked filters were probed with a 1∶1000 dilution of mouse anti-beta-actin monoclonal antibody (Sigma). Goat anti-mouse IgG conjugated with alkaline phosphatase (Sigma) was used as the secondary antibody. Membranes were then developed using the BCIP®/NBT Kit (Sigma) following the manufacturer's instructions. No endogenous alkaline phosphatase activity was detected when probing blots of BBMV proteins with streptavidin-AP or under direct exposure to NBT-BCIP. All blots were repeated in triplicate to ensure reproducibility of results.

## Supporting Information

Figure S1 Size distribution of assembled contigs.
(TIF)

Table S1 KEGG summary of *Chilo suppressalis* midgut sequences.
(XLS)

Table S2 Pfam domain search of *Chilo suppressalis* midgut sequences.
(XLS)

Table S3 Proteins identified by MADIL-TOF/TOF and de novo sequence analysis.
(DOC)
