# Correction: Exploring the Midgut Transcriptome and Brush Border Membrane Vesicle Proteome of the Rice Stem Borer, *Chilo suppressalis* (Walker)

**DOI:** 10.1371/annotation/46dce407-4b4f-476b-b405-8dff023e17da

**Published:** 2012-09-18

**Authors:** Weihua Ma, Zan Zhang, Chuanhua Peng, Xiaoping Wang, Fei Li, Yongjun Lin

There were errors in the Funding section. The correct funding information is: This research was supported by the Key Project for Breeding Genetic Modified Organisms (2011ZX08012-004). The funders had no role in study design, data collection and analysis, decision to publish, or preparation of the manuscript.

