# Supplementary figures and images for: Exploring the Midgut Transcriptome and Brush Border Membrane Vesicle Proteome of the Rice Stem Borer, Chilo suppressalis (Walker)
Source: PLoS One. 2012 May 29;7(5):e38151. doi: 10.1371/journal.pone.0038151 (PMC3362559; doi:10.1371/journal.pone.0038151)

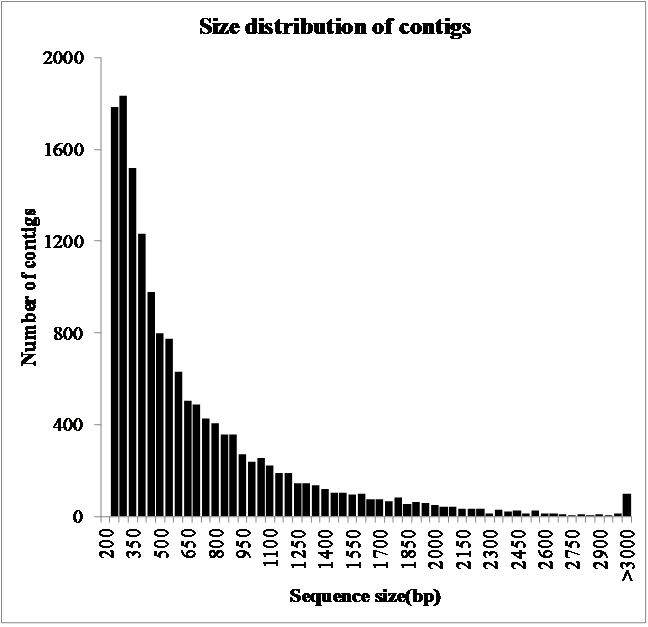

Supplement: Figure S1 — Size distribution of assembled contigs. (TIF) [file pone.0038151.s001.tif]
