# Supplementary material for: Exploring the Midgut Transcriptome and Brush Border Membrane Vesicle Proteome of the Rice Stem Borer, Chilo suppressalis (Walker)
Source: PLoS One. 2012 May 29;7(5):e38151. doi: 10.1371/journal.pone.0038151 (PMC3362559; doi:10.1371/journal.pone.0038151)
Supplement: Table S3 — Proteins identified by MADIL-TOF/TOF and de novo sequence analysis. (DOC) [file pone.0038151.s004.doc]

| Spot | Protein identified | Species matched | Database searched | Accession no. | Predicted MW/PI | Peptides Count^a^ | Protein score | E-value^b^ | Peptide sequence^c^ | Protein function |
| --- | --- | --- | --- | --- | --- | --- | --- | --- | --- | --- |
| 1 | Alpha spectrin | *Chilo suppressalis* | Translated transcriptome library |  | 278.3/5.08 | 7 | 56 | 3e-04 | QNQIEGQYDNLLALAR | Major constituent of the cytoskeletal network underlying the erythrocyte plasma membrane |
| 3 | Cry1Ac receptor | *Heliothis virescens* | NCBInr | gi|1063628 | 113.4/5.27 | 4 | 106 | 0.009 | AQIVNDVFQFAR | Acts as a receptor for Bthuringiensis Cry1Ac delta-endotoxin |
| 4 | Bicoid stability factor | *Chilo suppressalis* | Translated transcriptome library |  | 157.3/6.73 | 6 | 170 | 0.052 | CCGELVPEELPEQR | mRNA 3'-UTR binding |
| 6 | Gelsolin | *Chilo suppressalis* | Translated transcriptome library |  | 83.2/4.89 | 2 | 61 | 0.050 | YLDGGTESGFNQVEINAGAEKR | Actin binding |
| 7 | Myosin heavy chain variant A | *Chilo suppressalis* | Translated transcriptome library |  | 65.9/6.24 | 7 | 54 | 8e-06 | EEQAEADGTEDGEKVAK | ATP binding, actin binding, motor activity |
| 8 | Transitional endoplasmic reticulum ATPase TER94 | *Bombyx mori* | NCBInr | gi|112983322 | 89.8/5.30 | 16 | 306 | 3e-06 | VTQGFSGADLTEICQR | Nucleoside-triphosphatase activity |
| 9 | Translation elongation factor 2 | *Spodoptera exigua* | NCBInr | gi|28627569 | 95.6/6.14 | 18 | 144 | 4e-17 | LMEPVYLCEIQCPEVAVGGIYGVLNR | mRNA-binding protein, involved in translation elongation |
| 10 | Carbamoyl-phosphate synthase large chain | *Chilo suppressalis* | Translated transcriptome library |  | 150.6/5.73 | 4 | 50 | 3e-08 | LSGADVTLGVEMASTGEVACFGENRYEAYLK | ATP binding, carbamoyl-phosphate synthase activity |
| 12 | Succinate dehydrogenase complex, subunit A, flavoprotein (Fp) | *Chilo suppressalis* | Translated transcriptome library |  | 72.5/6.30 | 11 | 274 | 2e-07 | GSDWLGDQDAIHYMTR | Transferring electrons from succinate to ubiquinone |
| 13 | Fumarase | *Chilo suppressalis* | Translated transcriptome library |  | 53.5/8.82 | 11 | 207 | 8e-15 | EGHFPLVIWQTGSGTQSNMNTNEVIANR | Fumarate hydratase activity |
| 15 | Short-chain dehydrogenase | *Chilo suppressalis* | Translated transcriptome library |  | 32.6/6.39 | 4 | 45 | 1e-04 | GGVAVADYNSVVEGEK | Oxidoreductase activity |
| 16 | Short-chain dehydrogenase | *Chilo suppressalis* | Translated transcriptome library |  | 32.6/6.39 | 5 | 62 | 1e-06 | DKGGVAVADYNSVVEGEK | Oxidoreductase activity |
| 17 | Acyl-coa dehydrogenase | *Chilo suppressalis* | Translated transcriptome library |  | 69.1/8.17 | 14 | 282 | 1e-04 | CSNTTEVYYEDVKVPVR | acyl-CoA dehydrogenase activity, flavin adenine dinucleotide binding |
| 18 | Alpha-amylase | *Spodoptera frugiperda* | NCBInr | gi|27447982 | 51.8/6.38 | 7 | 142 | 0.006 | SGNEQQFANMVR | Cation binding; hydrolase activity, acting on glycosyl bonds |
| 19 | Endocytosis/signaling protein EHD1 | *Glossina morsitans morsitans* | NCBInr | gi|289740643 | 61.2/6.10 | 11 | 214 | 2e-10 | GEGIDAGYGEHDWICNRDK | GTP binding; GTPase activity; calcium ion binding |
| 20 | Putative achaete scute target 1, isoform A | *Drosophila melanogaster* | NCBInr | gi|24646379 | 61.7/5.98 | 14 | 252 | 3e-08 | GYDFTGVLEWFAERVDR | GTP binding; GTPase activity; calcium ion binding |
| 21 | 26S protease regulatory subunit-like protein | *Toxoptera citricida* | NCBInr | gi|54287934 | 49.4/5.35 | 17 | 289 | 2e-13 | SLDEEDIALLKTYGQGQWTINVK | ATP binding; nucleoside-triphosphatase activity; peptidase activity |
| 22 | Past-1 | *Chilo suppressalis* | Translated transcriptome library |  | 61.4/6.09 | 22 | 538 | 4e-08 | LFEDEEQDLFRDMQSLPR | [GTP binding](http://www.ebi.ac.uk/ego/DisplayGoTerm?id=GO:0005525),  [GTPase activity](http://www.ebi.ac.uk/ego/DisplayGoTerm?id=GO:0003924), [calcium ion binding](http://www.ebi.ac.uk/ego/DisplayGoTerm?id=GO:0005509) |
| 23 | Glutamate dehydrogenase | *Bombyx mori* | NCBInr | gi|114052462 | 61.9/8.36 | 7 | 236 | 3e-10 | ISGASEKDIVHSGLDYTMER | Oxidoreductase activity, involved in cellular amino acid metabolic process |
| 24 | Glutamate dehydrogenase | *Bombyx mori* | NCBInr | gi|114052462 | 61.9/8.36 | 10 | 308 | 4e-09 | GFIGPGVDVPAPDMGTGER | Oxidoreductase activity, involved in cellular amino acid metabolic process |
| 26 | Chaperonin | *Chilo suppressalis* | Translated transcriptome library |  | 59.1/5.62 | 12 | 101 | 3e-15 | DAGATLAICQWGFDDEANHLLLSSGLPAVR |  |
| 27 | ATP synthase | *Bombyx mori* | NCBInr | gi|114052278 | 59.8/9.21 | 8 | 382 | 9e-06 | TGAIVDVPVGEQILGR | Producing ATP from ADP |
| 28 | ATP synthase | *Bombyx mori* | NCBInr | gi|114052278 | 59.8/9.21 | 11 | 418 | 9e-06 | TGAIVDVPVGEQILGR | Producing ATP from ADP |
| 29 | ATP synthase | *Bombyx mori* | NCBInr | gi|114052278 | 59.8/9.21 | 21 | 791 | 9e-06 | TGAIVDVPVGEQILGR | Producing ATP from ADP |
| 30 | 4-Hydroxybutyrate CoA-transferase | *Chilo suppressalis* | Translated transcriptome library |  | 51.4/8.10 | 2 | 149 | 8e-06 | AHVQYVVTEQGIANLFGK | Acetyl-CoA metabolic process transferase activity |
| 31 | Isocitrate dehydrogenase | *Chilo suppressalis* | Translated transcriptome library |  | 38.7/8.30 | 6 | 189 | 4e-12 | TLYDNVDVVTIRENTEGEYSGIEHEIVDGVVQSIK | NAD or NADH binding, isocitrate dehydrogenase (NADP+) activity, magnesium ion binding |
| 32 | Elongation factor Tu | *Chilo suppressalis* | Translated transcriptome library |  | 51.0/8.47 | 11 | 436 | 6e-09 | ELDKPFLMPVESVHSIPGR | This protein promotes the GTP-dependent binding of aminoacyl-Trna to the A-site of ribosomes during protein biosynthesis. |
| 33 | Predicted: similar to citrate synthase | *Tribolium castaneum* | NCBInr | gi|91083623 | 51.7/8.65 | 4 | 112 | 6e-04 | SGQVVPGYGHAVLR | Transferase activity, transferring acyl groups |
| 34 | Mitochondrial processing peptidase beta subunit | *Chilo suppressalis* | Translated transcriptome library |  | 52.9/5.95 | 9 | 195 | 1e-06 | AASIENLCHSFQSFNTCYK | Metalloendopeptidase activity, zinc ion binding |
| 35 | Glutamate dehydrogenase | *Chilo suppressalis* | Translated transcriptome library |  | 61.4/8.39 | 7 | 294 | 2e-07 | ESNYHLLESVQESLERR | [binding](http://www.ebi.ac.uk/ego/DisplayGoTerm?id=GO:0005488), [oxidoreductase activity](http://www.ebi.ac.uk/ego/DisplayGoTerm?id=GO:0016491) |
| 36 | Elongation factor Tu | *Chilo suppressalis* | Translated transcriptome library |  | 51.0/8.47 | 11 | 293 | 6e-09 | ELDKPFLMPVESVHSIPGR | This protein promotes the GTP-dependent binding of aminoacyl-Trna to the A-site of ribosomes during protein biosynthesis. |
| 37 | Enolase | *Bombyx mori* | NCBInr | gi|148298800 | 47.2/5.62 | 5 | 143 | 9e-07 | VNQIGSVTESIDAHLLAK | Magnesium ion binding; phosphopyruvate hydratase activity |
| 38 | Isocitrate dehydrogenase | *Chilo suppressalis* | Translated transcriptome library |  | 38.7/8.30 | 6 | 184 | 2e-08 | ENTEGEYSGIEHEIVDGV | NAD or NADH binding, isocitrate dehydrogenase (NADP+) activity, magnesium ion binding |
| 42 | cGmp-dependent protein kinase | *Chilo suppressalis* | Translated transcriptome library |  | 109.7/6.89 | 4 | 40 | 2e-05 | NIIYRDLKPENLMLDK | ATP binding, Cgmp-dependent protein kinase activity |
| 44 | ATP-dependent RNA helicase | *Chilo suppressalis* | Translated transcriptome library |  | 78.6/5.66 | 6 | 56 | 0.021 | QSSGGILVATDVAARGL | ATP binding, ATP-dependent helicase activity, RNA binding |
| 45 | Dynamin | *Chilo suppressalis* | Translated transcriptome library |  | 81.3/6.34 | 8 | 52 | 6e-09 | TLDSIHPLVGLTRMDILTAIR | GTP binding, GTPase activity |
| 47 | Bicoid stability factor | *Chilo suppressalis* | Translated transcriptome library |  | 157.3/6.73 | 5 | 138 | 0.052 | CCGELVPEELPEQR | Mrna 3’-UTR binding |
| 48 | Insecticidal Bt toxin receptor APN2 | *Plutella xylostella* | NCBInr | gi|1063628 | 107.3/5.33 | 2 | 88 | 7e-14 | NDQIASPFWASGATENWGLVTYR | metallopeptidase activity, receptor activity, zinc ion binding |
| 49 | Methylcrotonoyl-coenzyme A carboxylase 1 alpha | *Chilo suppressalis* | Translated transcriptome library |  | 77.8/6.10 | 3 | 54 | 0.17 | VAAGEPLPLTQEEIIR | ATP binding, biotin binding, ligase activity |
| 51 | Myosin-IB | *Chilo suppressalis* | Translated transcriptome library |  | 119.4/9.07 | 4 | 40 | 8e-06 | LLQTNPLLEAFGNAKTNR | [ATP binding](http://www.ebi.ac.uk/ego/DisplayGoTerm?id=GO:0005524), [motor activity](http://www.ebi.ac.uk/ego/DisplayGoTerm?id=GO:0003774) |
| 53 | Muscle myosin heavy chain | *Chilo suppressalis* | Translated transcriptome library |  | 224.6/5.75 | 7 | 53 | 8e-06 | EEQAEADGTEDGEKVAK | Motor protein, muscle protein, myosin |
| 55 | Alpha-tubulin | *Chilo suppressalis* | Translated transcriptome library |  | 50.1/4.97 | 16 | 221 | 2e-11 | QLFHPEQLITGKEDAANNYAR | The major constituent of microtubules, binding to GTP |
| 56 | Eukaryotic translation termination factor 1 | *Chilo suppressalis* | Translated transcriptome library |  | 49.2/5.49 | 6 | 42 | 3e-10 | SLEMARGNGTSMISLIIPPK | [Translation release factor activity, codon specific](http://www.ebi.ac.uk/ego/DisplayGoTerm?id=GO:0016149) |
| 57 | Elongation factor 1 gamma | *Bombyx mori* | NCBInr | gi|112983898 | 48.6/5.83 | 7 | 81 | 9e-06 | VFMSCNLITGMFQR | Translation elongation factor activity, involved in protein biosynthesis |
| 60 | Enolase | *Oncometopia nigricans* | NCBInr | gi|53830714 | 46.8/5.92 | 7 | 193 | 9e-12 | LAMQEFMILPTGASSFTEAMK | Magnesium ion binding; [phosphopyruvate hydratase activity](http://www.ebi.ac.uk/ego/DisplayGoTerm?id=GO:0004634) |
| 61 | Heat shock protein 60 | *Chilo suppressalis* | NCBInr | gi|253993196 | 61.1/5.69 | 6 | 287 | 7e-06 | AAVEEGIVPGGGSALLR | [ATP binding](http://www.ebi.ac.uk/ego/DisplayGoTerm?id=GO:0005524); unfolded protein binding, involved in stress response |
| 62 | Heat shock cognate 70 | *Aedes aegypti* | NCBInr | gi|94468818 | 72.3/5.06 | 12 | 462 | 2e-07 | VTHAVVTVPAYFNDAQR | [ATP binding](http://www.ebi.ac.uk/ego/DisplayGoTerm?id=GO:0005524); nucleotide binding, involved in stress response |
| 63 | Heat shock cognate protein 70 | *Chilo suppressalis* | NCBInr | gi|74271761 | 71.6/5.24 | 8 | 167 | 2e-08 | TVQNAVITVPAYFNDSQR | [ATP binding](http://www.ebi.ac.uk/ego/DisplayGoTerm?id=GO:0005524); nucleotide binding, involved in stress response |
| 64 | Heat shock cognate protein 70 | *Chilo suppressalis* | NCBInr | gi|74271761 | 71.6/5.24 | 11 | 365 | 2e-08 | TVQNAVITVPAYFNDSQR | [ATP binding](http://www.ebi.ac.uk/ego/DisplayGoTerm?id=GO:0005524); nucleotide binding, involved in stress response |
| 65 | Chaperonin subunit 6a zeta | *Chilo suppressalis* | Translated transcriptome library |  | 57.4/6.59 | 9 | 180 | 4e-13 | ASTAQDDATGDGTTSTVLLIGELLK | ATP binding, unfolded protein binding |
| 66 | Glutamate dehydrogenase, isoform F | *Drosophila melanogaster* | NCBInr | gi|45553475 | 61.4/8.58 | 13 | 427 | 5e-07 | GFIPGVDVPAPDMGTGER | [Glutamate dehydrogenase [NAD(P)+] activity](http://www.ebi.ac.uk/ego/DisplayGoTerm?id=GO:0004353), involved in [cellular amino acid metabolic process](http://www.ebi.ac.uk/ego/DisplayGoTerm?id=GO:0006520) |
| 68 | Dihydrolipoamide succinyltransferase | *Chilo suppressalis* | Translated transcriptome library |  | 43.4/5.65 | 7 | 161 | 0.12 | NVQNMTYADIELTVAGLAEK | Tricarboxylic acid cycle dihydrolipoyllysine-residue succinyltransferase activity |
| 69 | Hsc70/Hsp90-organizing protein HOP | *Chilo suppressalis* | Translated transcriptome library |  | 62.7/6.14 | 6 | 113 | 4e-05 | LAAFDIGLKDCDQCCK | Binding |
| 71 | Carbamoyl-phosphate synthase large chain | *Chilo suppressalis* | Translated transcriptome library |  | 150.6/5.73 | 3 | 58 | 0.069 | VILGIPVETVNVLAGCGK | ATP binding, carbamoyl-phosphate synthase activity |
| 72 | 26S proteasome regulatory complex subunit p48B | *Chilo suppressalis* | Translated transcriptome library |  | 48.5/5.75 | 12 | 214 | 3e-09 | FVVDLADSVAPTDIEEGMR | ATP binding, ATPase activity, uncoupled, endopeptidase activity |
| 74 | EH domain-containing protein 1 | *Camponotus floridanus* | NCBInr | gi|307167685 | 109.2/7.95 | 15 | 132 | 0.03 | LFEDEEQDLFR | GTP binding, GTPase activity, acid phosphatase activity, calcium ion binding |
| 75 | Past-1 | *Chilo suppressalis* | Translated transcriptome library |  | 61.4/6.09 | 15 | 210 | 1e-04 | MQETLANHDFSKFHPLKPK | [GTP binding](http://www.ebi.ac.uk/ego/DisplayGoTerm?id=GO:0005525),  [GTPase activity](http://www.ebi.ac.uk/ego/DisplayGoTerm?id=GO:0003924), [calcium ion binding](http://www.ebi.ac.uk/ego/DisplayGoTerm?id=GO:0005509) |
| 76 | Adenylsulfate kinase | *Chilo suppressalis* | Translated transcriptome library |  | 69.8/5.87 | 11 | 176 | 8e-11 | KPVLLLHPLGGWTKDDDVPLEVR | ATP binding, adenylylsulfate kinase activity, sulfate adenylyltransferase (ATP) activity |
| 78 | Esterase | *Chilo suppressalis* | Translated transcriptome library |  | 63.3/8.43 | 4 | 40 | 0.001 | LLPVMVYFHGGGYFAGSSSLSLYGPNYLISK | Hydrolase activity |
| 79 | Mitochondrial precursor protein import receptor | *Chilo suppressalis* | Translated transcriptome library |  | 64.7/5.49 | 5 | 78 | 0.39 | AISLYNEAIEACPPDRPVDLATFYQNR | Protein binding |
| 80 | Chaperonin containing t-complex polypeptide 1 beta subunit | *Chilo suppressalis* | Translated transcriptome library |  | 57.6/6.32 | 12 | 157 | 3e-19 | QLIYNYPEQLFADAGVMAIEHADFDGIER | ATP binding, unfolded protein binding |
| 82 | Actin | *Chorthippus parallelus* | NCBInr | gi|283483333 | 37.9/5.46 | 14 | 470 | 3e-08 | DLYANTVLSGGTTMYPGI | Cytoskeleton structure, cell mobility, chromosome movement and muscle contraction. |
| 83 | ARP1 actin-related protein 1-like protein A | *Chilo suppressalis* | Translated transcriptome library |  | 42.7/6.62 | 9 | 54 | 1e-06 | ACYLSPNPLKEETLDTER | ATP binding |
| 84 | Elongation factor Tu | *Chilo suppressalis* | Translated transcriptome library |  | 51.0/8.47 | 10 | 354 | 6e-09 | ELDKPFLMPVESVHSIPGR | This protein promotes the GTP-dependent binding of aminoacyl-Trna to the A-site of ribosomes during protein biosynthesis. |
| 85 | Flotillin-1 | *Chilo suppressalis* | Translated transcriptome library |  | 45.8/5.91 | 17 | 151 | 1e-06 | LTGEVLSIVQCIPDLVKGVTGVDISK | Regulates caveolin-1 level by preventing its lysosomal degradation in intestinal epithelial cells |
| 86 | Electron transfer flavoprotein-ubiquinone oxidoreductase | *Chilo suppressalis* | Translated transcriptome library |  | 66.1/6.08 | 9 | 78 | 0.001 | IPIPVFPGLPMYNHGNYVVR | Electron carrier activity, electron-transferring-flavoprotein dehydrogenase activity |
| 88 | 2-hydroxyphytanoyl-CoA lyase | *Chilo suppressalis* | Translated transcriptome library |  | 64.8/7.11 | 1 | 54 | 0.12 | SEVPVHSDIKPFVEALTR | Lyase activity, magnesium ion binding, thiamine pyrophosphate binding |
| 90 | Succinate dehydrogenase | *Chilo suppressalis* | Translated transcriptome library |  | 72.1/6.35 | 7 | 256 | 1e-11 | SYFSCTSAHTCTGDGTAMAARAGLQ | Electron carrier activity, flavin adenine dinucleotide binding, oxidoreductase activity, acting on the CH-CH group of donors |
| 91 | Glutamate dehydrogenase | *Chilo suppressalis* | Translated transcriptome library |  | 61.9/8.36 | 7 | 288 | 2e-07 | ESNYHLLESVQESLERR | Oxidoreductase activity, involved in cellular amino acid metabolic proces |
| 92 | Chaperonin containing t-complex polypeptide 1 beta subunit | *Chilo suppressalis* | Translated transcriptome library |  | 57.6/6.32 | 9 | 60 | 3e-10 | VQDDEVGDGTTSVTVLAAELLR | ATP binding, unfolded protein binding |
| 93 | Metallopeptidase | *Chilo suppressalis* | Translated transcriptome library |  | 43.8/7.57 | 8 | 54 | 2e-06 | ATLETYEVYAMDVLVSTGEGVGR | Involved in cellular process |
| 94 | Eukaryotic translation initiation factor 3 subunit I | *Chilo suppressalis* | Translated transcriptome library |  | 36.9/5.71 | 6 | 61 | 4e-11 | TERPVNSAALSPILDHVVLGGGQDAMEVTTTSTR | Nucleotide binding, translation initiation factor activity |
| 96 | Enolase | *Chilo suppressalis* | Translated transcriptome library |  | 46.8/5.92 | 3 | 90 | 3e-07 | TPIQIVGDDLTVTNPKR | Magnesium ion binding; [phosphopyruvate hydratase activity](http://www.ebi.ac.uk/ego/DisplayGoTerm?id=GO:0004634) |
| 99 | H+ transporting ATP synthase beta subunit | *Heliconius cydno cordula* | NCBInr | gi|298400745 | 37.5/4.96 | 15 | 824 | 1e-05 | VALVYGQMNEPPGAR | Produces ATP from ADP in the presence of a proton gradient across the membrane |
